# Correction: Aerosols transmit prions to immunocompetent and immunodeficient mice

**DOI:** 10.1371/journal.ppat.1005463

**Published:** 2016-02-12

**Authors:** Johannes Haybaeck, Mathias Heikenwalder, Britta Klevenz, Petra Schwarz, Ilan Margalith, Claire Bridel, Kirsten Mertz, Elizabeta Zirdum, Benjamin Petsch, Thomas J. Fuchs, Lothar Stitz, Adriano Aguzzi

The authors would like to correct Figs 3, 5 and 6. In Fig 3, an error was introduced during the preparation of the figure for publication. Images from the brain of a mouse presented in Fig 4A were inserted into Fig 3A. The corrected version of Fig 3, containing pictures of a correct and representative *JH* ^*-/-*^ mouse that had been exposed to prion infectivity containing aerosols, can be seen here. The authors also wish to clarify that the original blots for Figs 5E and 6D contained redundant lanes which they had removed from the images while preparing the figures. The authors now provide corrected versions of Figs 5E and 6D with appropriate marks showing the removal of the redundant lane. The uncropped original blots for Figs 5E and 6D are shown as supporting information in S1 File (for Fig 5E) and S2 File (for Fig 6D).

**Fig 3:**
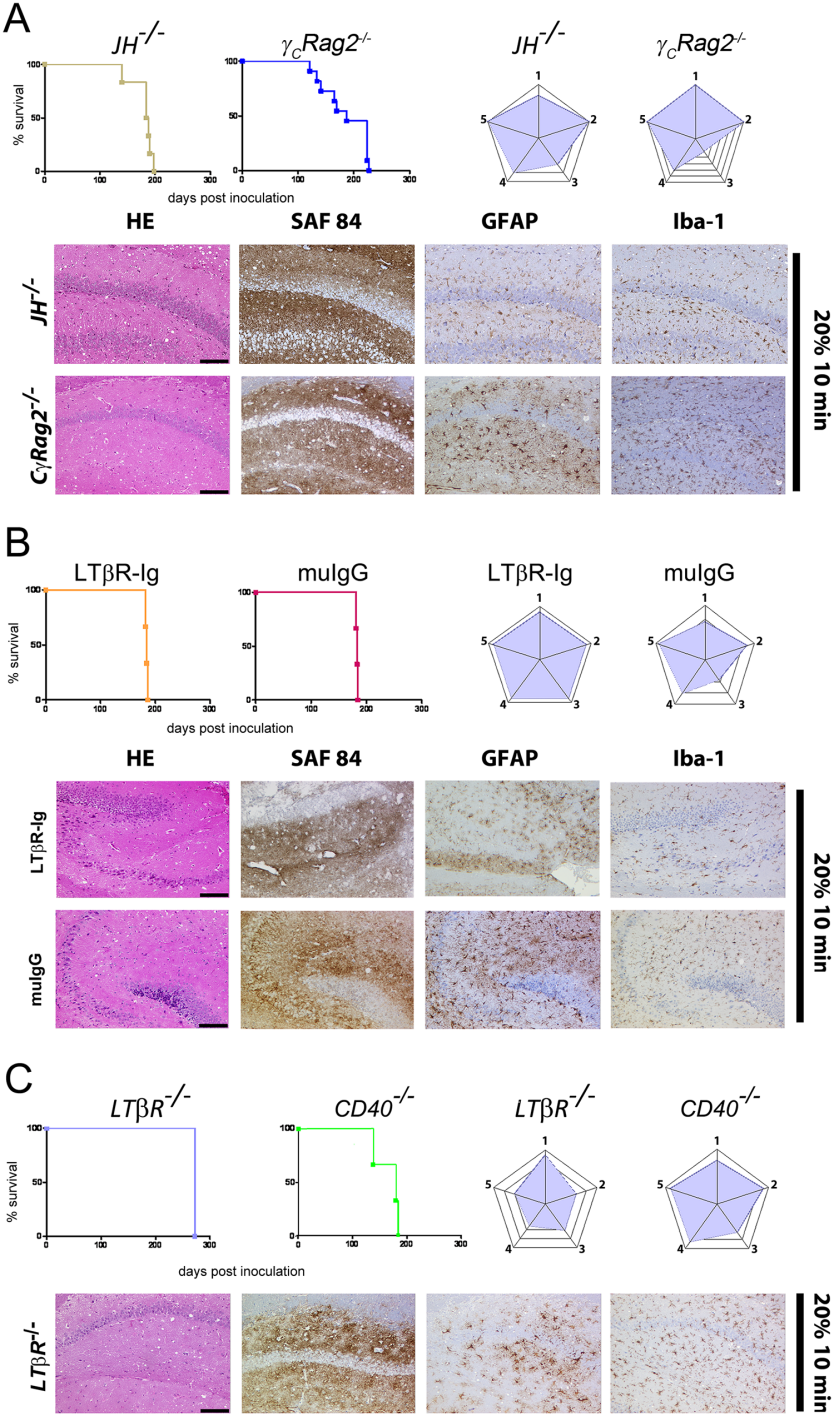
Prion transmission through aerosols in immunocompromised mice. Survival curves, lesion severity score analysis (radar plots), and representative histopathological micrographs of mice with genetically or pharmacologically impaired components of the immune system (*JH* ^−/−^, *γ*_*C*_ *Rag2* ^−/−^) (A), 129Sv mice treated with LTβR-Ig or with muIgG (B), *LT*β*R* ^−/−^ and *CD40* ^−/−^ mice (C). All mice were exposed for 10 min to aerosolized 20% IBH. Stain code: HE (spongiosis, gliosis, neuronal cell loss), SAF84 (PrP^Sc^ deposits), GFAP (astrogliosis) and Iba-1 (microglial activation) as in Fig 1H. Scale bars: 100 μm.

**Fig 5:**
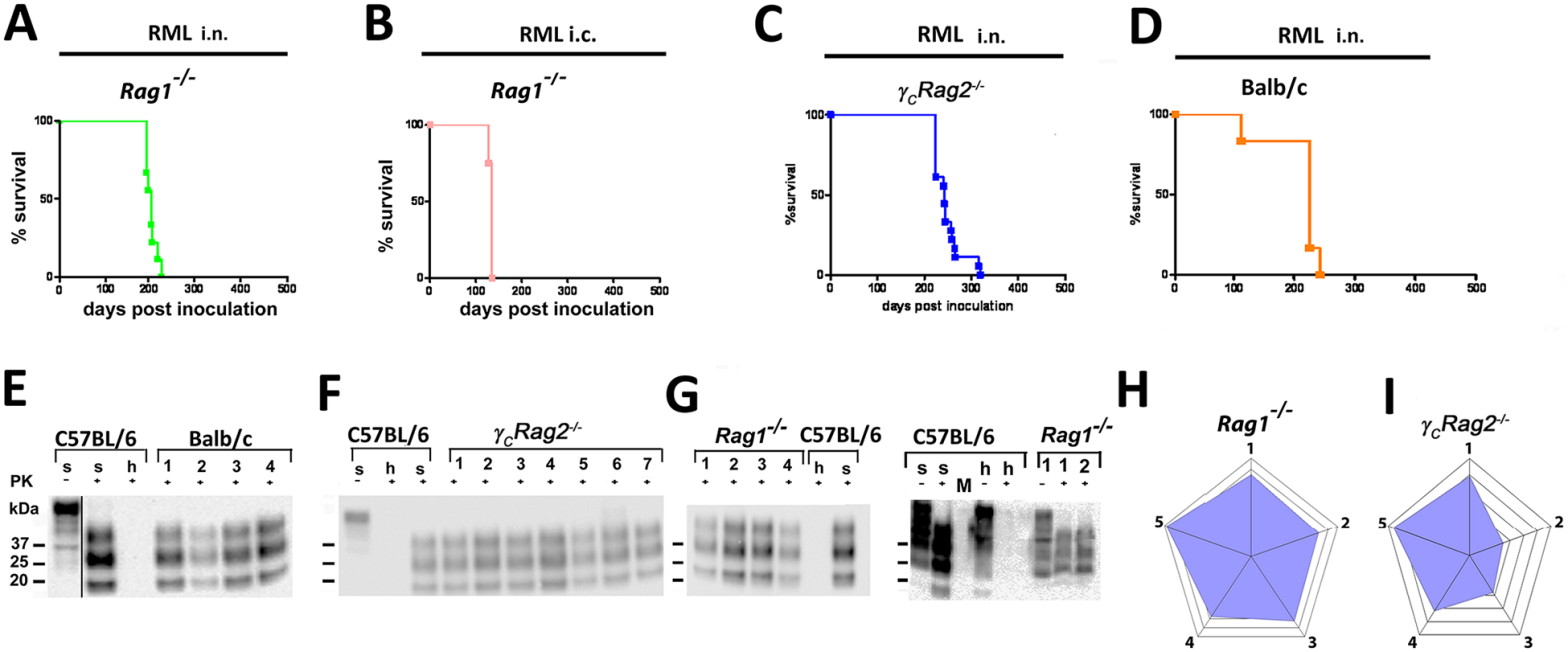
Prion transmission by intranasal instillation. (A) *Rag1* ^−/−^ mice intranasally inoculated with RML6 0.1%, (B) *Rag1* ^−/−^ mice i.c. inoculated with 3×10^5^ LD_50_, (C) *γ*_*C*_ *Rag2* ^−/−^ mice intranasally inoculated with 4×10^5^ LD_50_ or (D) Balb/c mice intranasally inoculated with 4×10^5^ LD_50_ scrapie prions are shown. Survival curves (A–D) and respective Western blots (E–G) are indicative of efficient prion neuroinvasion. (E) Black line indicates crop marks; uncropped blots are shown in S1 File. Brain homogenates were analyzed with (+) and without (−) previous proteinase K (PK) treatment as indicated. Brain homogenates derived from a terminally scrapie-sick and a healthy C57BL/6 mouse served as positive and negative controls (s: sick; h: healthy), respectively. Molecular weights (kDa) are indicated on the left side of the blots. (H and I) Histopathological lesion severity score described as radar blot (astrogliosis, spongiform change and PrP^Sc^ deposition) in 5 brain regions of both mouse lines exposed to prion aerosols. Numbers correspond to the following brain regions: (1) hippocampus, (2) cerebellum, (3) olfactory bulb, (4) frontal white matter, (5) temporal white matter.

**Fig 6:**
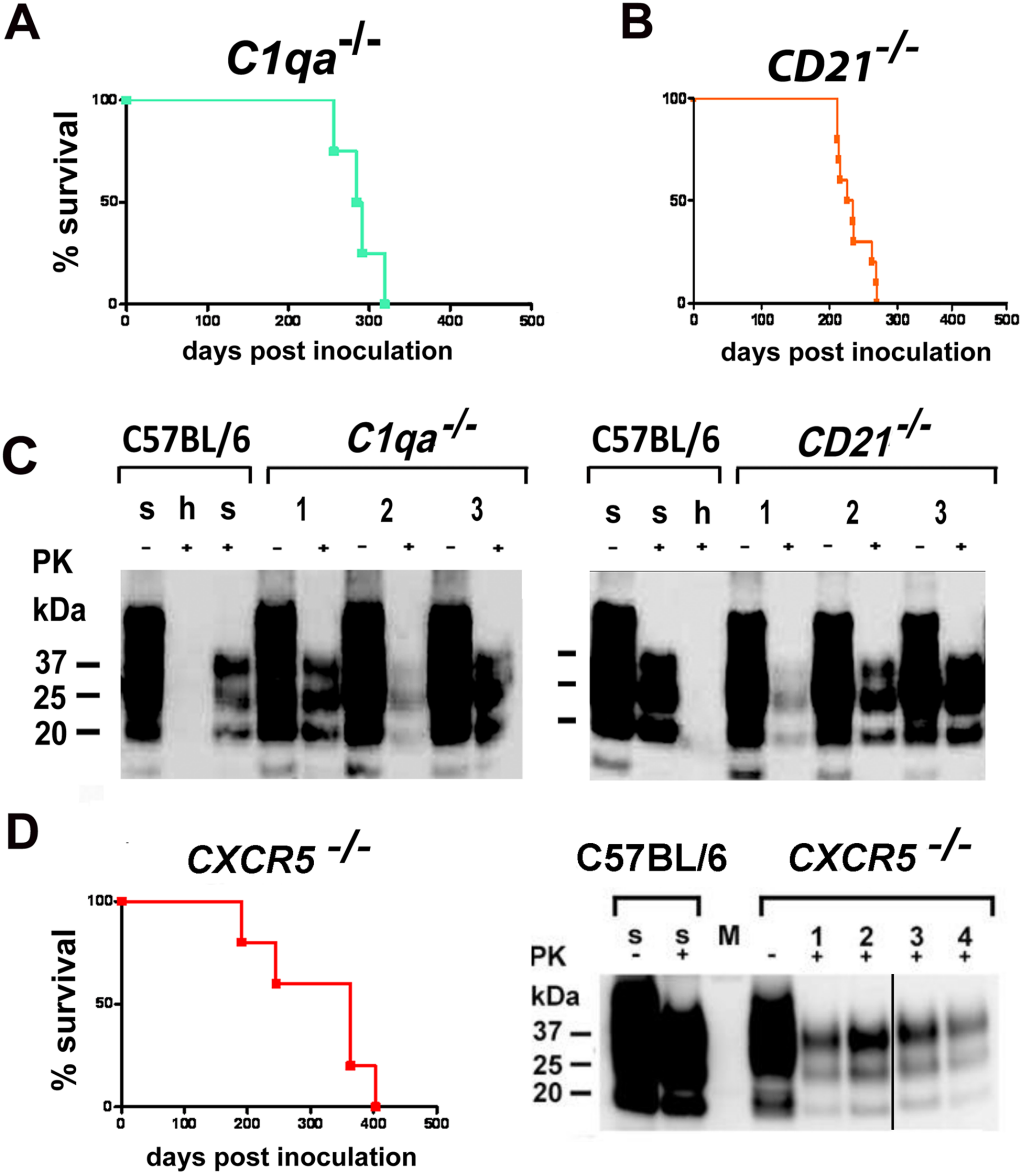
Intranasal prion transmission in immnunodeficient mice. All mice were intranasally inoculated with 3×10^5^ LD_50_ prions. (A) *C1q*a^−/−^ mice intranasally inoculated and (B) *CD21* ^−/−^ mice intranasally inoculated are shown. Survival curves illustrate survival after intranasal prion challenge. Respective Western blots of *C1qa* ^−/−^ mice intranasally inoculated (C, left panel) and of *CD21* ^−/−^ mice intranasally inoculated (C, right panel) are shown. Survival curves of *CXCR5* ^−/−^ mice intranasally inoculated are shown (D, left panel). Respective Western blot of *CXCR5* ^−/−^ mice intranasally inoculated is presented (D, right panel). Brain homogenates were analyzed with (+) and without (−) previous proteinase K (PK) treatment as indicated. (D, right panel) Black line indicates crop marks; uncropped blots are shown in S1 File. Controls and legends are as in Fig 5.

The corrected versions of Figs 3, 5 and 6 and the original uncropped blots for Figs 5E and 6D can be viewed below. The authors confirm that these changes do not alter their findings.

## Supporting Information

S1 File Uncropped blots for Fig 5E. Individual blots at various exposure times are shown.
(TIF)

S2 File Uncropped blots for Fig 6D. Individual blots at various exposure times are shown.
(TIF)
