# Supplementary figures and images for: Correction: Aerosols transmit prions to immunocompetent and immunodeficient mice
Source: PLoS Pathog. 2016 Feb 12;12(2):e1005463. doi: 10.1371/journal.ppat.1005463 (PMC4752327; doi:10.1371/journal.ppat.1005463)

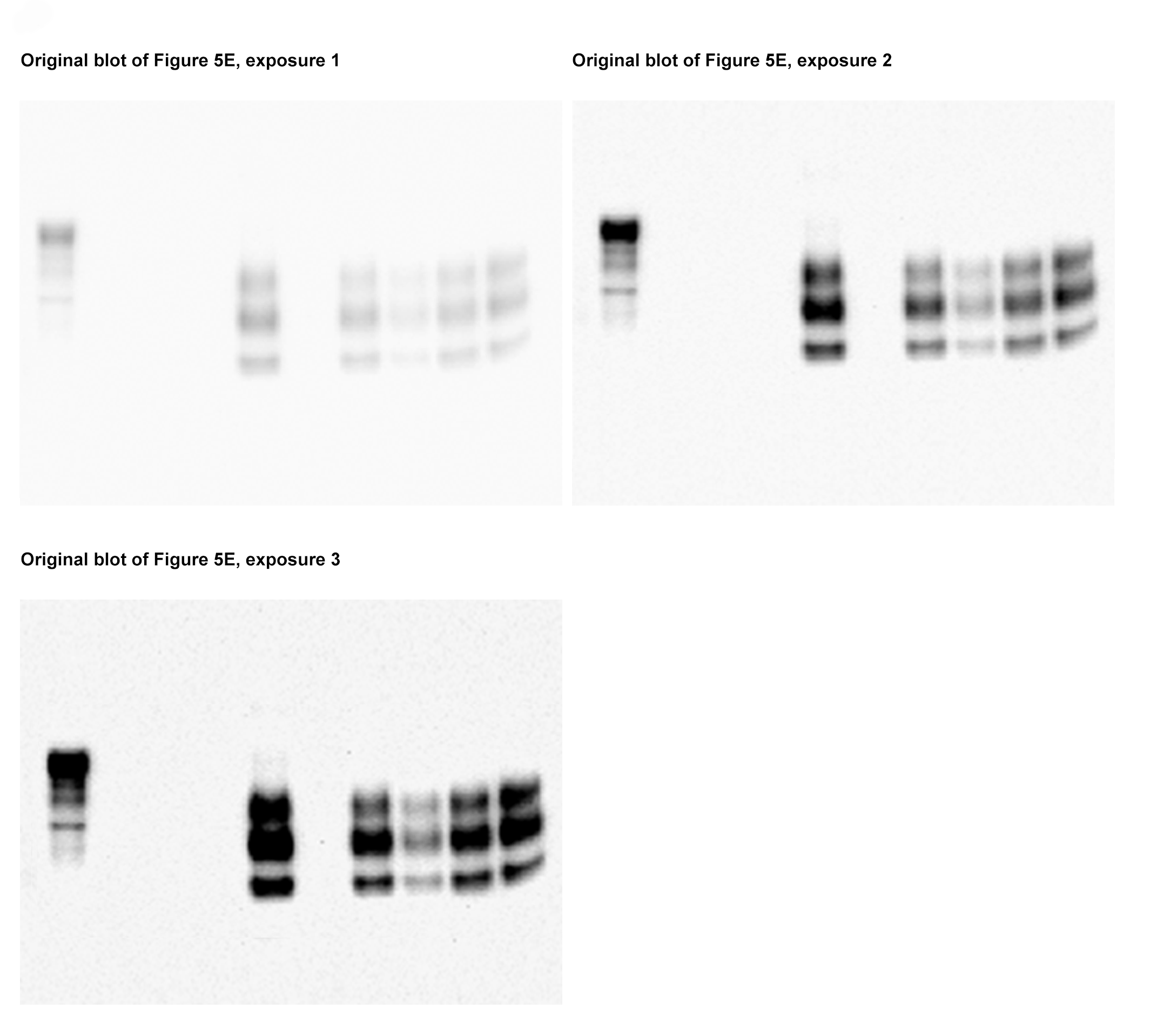

Supplement: S1 File — Individual blots at various exposure times are shown. (TIF) [file ppat.1005463.s001.tif]

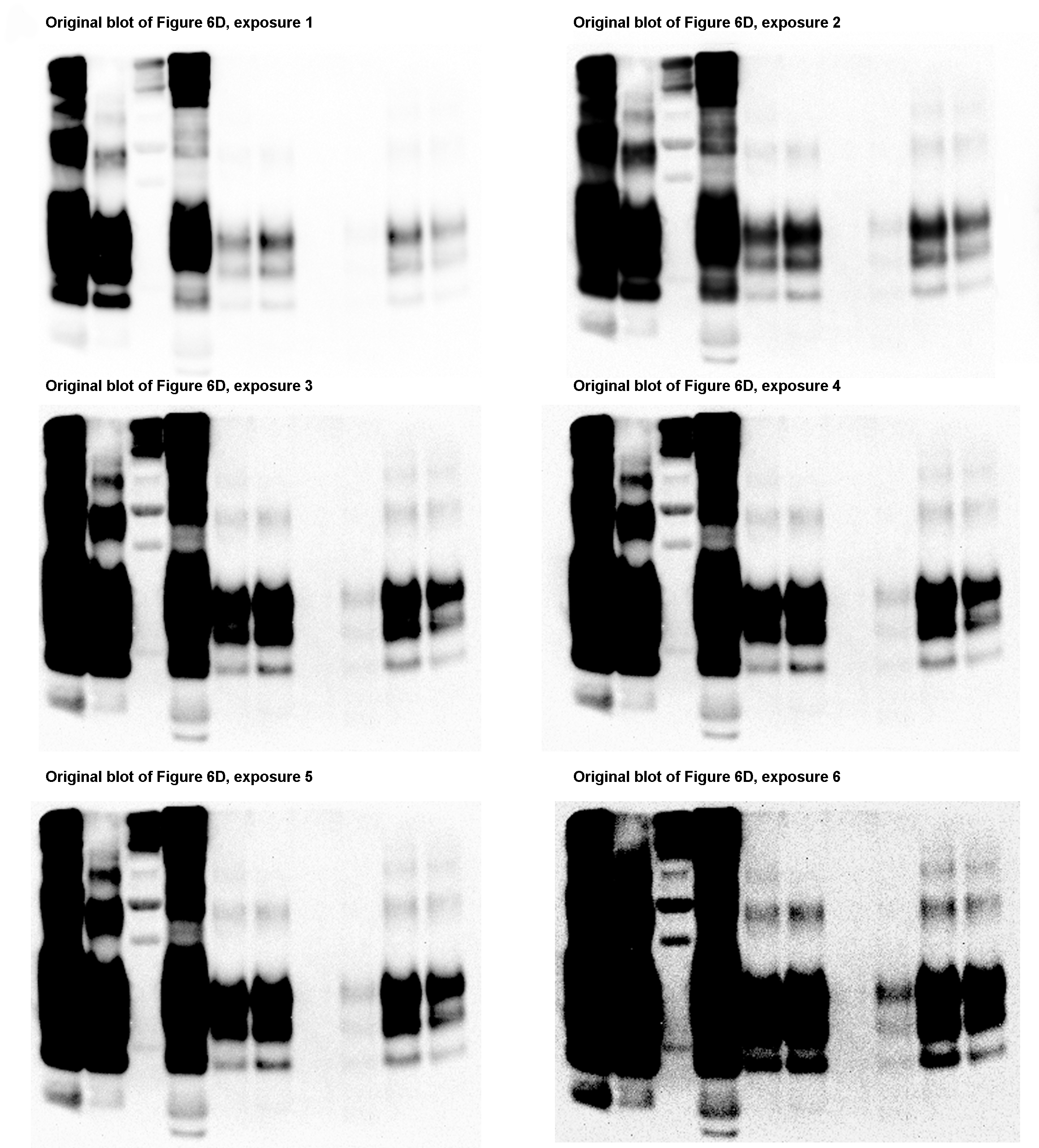

Supplement: S2 File — Individual blots at various exposure times are shown. (TIF) [file ppat.1005463.s002.tif]
